# Detection of *Schistosoma mansoni* DNA using polymerase chain reaction from serum and dried blood spot card samples of an adult population in North-western Tanzania

**DOI:** 10.1186/s40249-021-00798-4

**Published:** 2021-02-23

**Authors:** Antje Fuss, Humphrey D. Mazigo, Andreas Mueller

**Affiliations:** 1 Medical Mission Institute, Hermann-Schell-Str. 7, 97074 Wuerzburg, Germany; 2 Department of Medical Parasitology and Entomology, School of Medicine, Catholic University of Health and Allied Sciences, P.O. Box 1464, Mwanza, Tanzania; 3 Department of Tropical Medicine, Klinikum Wuerzburg Mitte gGmbH, Medical Mission Hospital, Wuerzburg, Germany

**Keywords:** *Schistosoma mansoni*, Real-time PCR, Circulating DNA, Dried blood spots, Tanzania

## Abstract

**Background:**

Real-time polymerase chain reaction (PCR) is a sensitive and specific method for diagnosing schistosomiasis. However, this method should be performed in a laboratory, usually located distant from the sample collection site. Therefore, it is important to have fast sampling preservation methods, which allow simple transport prior to DNA extraction and amplification. The aim of this study was to verify if blood samples applied to filter paper are suitable for analysis of *Schistosoma mansoni* DNA by real-time PCR.

**Methods:**

A cross-sectional study was conducted among 100 study participants aged 17 to 70 years in a fishing village on the southern shore of Lake Victoria, Tanzania. Serum samples and ethylenediaminetetraacetic acid (EDTA)-anticoagulated whole blood for preparation of dried blood spots (DBS) were collected to test for *Schistosoma mansoni* infection by real-time PCR. A combined diagnostic reference of positive results of serum-based real-time PCR and the Kato-Katz (KK) method was used for analysis. Sensitivity and negative predictive value (NPV) were calculated. The Wilcoxon signed-rank test was chosen to compare the mean cycle threshold (Ct) values from serum and DBS.

**Results:**

According to the reference, 92.5% *S. mansoni* positive samples were determined. The serum-based real-time PCR performed excellently with 95.4% sensitivity, whereas the DBS-based real-time PCR showed a low sensitivity (45.4%). The Ct-values were significantly higher in DBS (median: 37.3) than in serum samples (median: 27.5, *P* < 0.001), reflecting a lower parasite-specific DNA load on the filter cards. With increasing egg counts, an increase in sensitivity was observed for all methods. The POC-CCA test and the serum-based real-time PCR showed a sensitivity of 100% for medium and severe infections. The DBS real-time PCR showed a sensitivity of only 85.7% even for severe infections.

**Conclusions:**

DBS-based real-time PCR did not provide good results in our study and therefore should not be recommended or must be tested concerning temperature of storage, storage duration, use of different filter papers and extraction methods before it is used in future studies. In contrast, our results showed that the POC-CCA test is a sensitive and precise test for detecting *S. mansoni* infections  .

## Background

Schistosomiasis is a major neglected tropical disease which leads to significant economic and public health consequences, particularly in rural communities [1, 2]. The disease is caused by trematodes of the genus *Schistosoma* [3]. In sub-Saharan Africa, the main burden of the disease is caused by two human schistosome species, *Schistosoma mansoni* and *S. haematobium* [4]. In endemic countries mass drug administration (MDA) campaigns with praziquantel (PZQ) have been often performed to reduce the morbidity [5]. This has led to lower infection intensities despite high parasite transmission rates. In these situations, but also under low prevalence and post-treatment conditions, highly sensitive and specific diagnostic tests are necessary [6, 7]. The most commonly used diagnostic methods for the detection of *S. mansoni*, the Kato-Katz thick smears technique (KK) for the microscopic identification of eggs in faeces and the rapid diagnostic test for the detection of circulating cathodic antigen (POC-CCA), lack sensitivity particularly in low intensity infections[8]. Due to the diagnostic accuracy, and the higher sensitivity, including the ability to detect early pre-patent infections, polymerase chain reaction (PCR)-based methods for detection of parasite DNA have moved into the focus of research [8–11]. Numerous studies have shown that different types of samples, such as blood [9, 12, 13], urine [14–16], stool [9, 17], infective snail tissue [18, 19] and cercaria in contaminated water [20], are suitable for the detection of schistosome DNA with molecular techniques. In the reviews of Weerakoon et al. [8, 21] all the different diagnostic methods were presented in great detail. Considering that the result of molecular diagnostic methods depends directly on the quantity and quality of the DNA used, the type of sample, sample conservation, sample storage as well as DNA extraction from these samples play a central role [16, 22–24]. The samples usually have to be transported to a central laboratory for processing and cannot be examined directly in the field [8]. Depending on the nature of the sample, this may require feasible and rapid preservation of the DNA, for example stabilisation of DNA in urine with special urine preservation reagents [25], freezing serum [26] or preserving DNA on dried blood spots (DBS) [27]. The use of DBS has the advantage that no cold chain is required for the transport and storage of the samples [28]. In addition, no venipuncture is required for the preparation, only blood from the fingertip [29].

The objective of this study was to test the usefulness of dried blood on DBS in comparison to frozen serum samples for molecular detection of *S. mansoni* DNA using real-time PCR in diagnosing cases of schistosomiasis due to *S. mansoni* in a high-prevalence area.

## Materials and methods

### Study area and population

A cross-sectional study was conducted in March 2018 in the community of Kayenze, a fishing village in Ilemela district on the southern shore of Lake Victoria in north-western Tanzania. The majority of the inhabitants depend on the lake for domestic and economic activities including fishing, farming, washing, bathing, cooking, drinking and recreation. The region is endemic for *S. mansoni* [30–32] and the high occupational exposure keeps the intensity of *S. mansoni* infection high in adulthood [33]. In the study by Mazigo et al. a prevalence of 54.2% and a mean value of 202 eggs per gram stool (EPG) was found in the same region [30, 31]. Annual MDA using praziquantel and albendazole against helminth infections in this village is school-based, targets school children and not adults [30]. The study included only participants between the ages of 17 and 70 years who provided written informed consent.

### Collection and preparation of blood samples

One hundred participants were assessed for infection with *S. mansoni*. Serum samples were obtained after centrifugation of coagulated blood samples at 1200×*g* for 5 min. The supernatant was centrifuged for a further 15 min at 3000×*g*. Both centrifugation steps were performed at ambient temperature. The resulting supernatant was pipetted into 2 ml tubes and stored and transported in special thermal packaging at − 20 °C. Dried blood spots were prepared by dropping 125 µl ethylenediaminetetraacetic acid (EDTA)-anticoagulated whole blood onto Whatman™ FTA™ Classic Cards (GE Healthcare Life Sciences, Piscataway, NJ, USA). Cards were dried away from direct sunlight, placed into individual zip bags with desiccant three hours after preparation, and stored at room temperature until processing [34, 35].

### Microscopic examination

Stool samples were evaluated for the presence of *S. mansoni* eggs by using the quantitative KK thick smear technique. For the KK method two thick smears were prepared from different parts of a single stool sample using a template of 41.7 mg (Vestergaard Frandsen, Lausanne, Switzerland), following a standard protocol [36, 37]. After 24 h, the smears were independently examined for *S. mansoni* eggs by two experienced laboratory technicians of the National Institute for Medical Research (NIMR) laboratory. For quality assurance, 10% of the negative and positive KK thick smears were re-examined by a third laboratory technician.

### Point-of-care circulating cathodic antigen (POC-CCA) urine rapid diagnostic test

Urine samples were tested for circulating cathodic antigen (CCA) of *Schistosoma* by the POC-CCA cassette test according to the protocol described by the manufacturer and noted as positive or negative (Rapid Diagnostics, Pretoria, South Africa). Trace readings were considered as positive test results.

### DNA extraction from serum samples

DNA extractions from 2 ml serum were performed using the QIAamp Circulating Nucleic Acid Kit according to the manufacturer’s suggestions (Qiagen, Hilden, Germany). DNA was stored at -20 °C after extraction.

### DNA extraction from dried blood spot cards

DNA extraction from the DBS was performed approximately 4 months after sample collection. Harris Uni-Core puncher (Qiagen, Hilden, Germany) was used to punch out circles (6 mm in diameter) of the DBS. Between samples, the Harris Uni-Core was cleaned as described previously [38]. Eight paper discs from each sample were punched out and distributed to four 1.5 ml Eppendorf tubes containing 200 μl PBS each. Tubes were placed on a rotator at room temperature overnight (16–18 h, 300 rpm). The entire PBS solution from the four tubes of the same sample was transferred to one Qiagen QIAmp 2 ml column tube. DNA was precipitated and concentrated using the QIAmp DNA Blood Mini Kit (Qiagen, MD) according to the manufacturer's protocol.

### Amplification by real-time PCR

Detection of cell-free *S. mansoni* DNA samples was performed according to a previously published protocol [39] using a set of primers and probes complementary to a 121 bp tandem repeat sequence of *S. mansoni* strain SM 1–7 (GenBank accession number: M61098) described by Hamburger et al. [40]. Primer sequences were: Sm FW 5′-CCG ACC AAC CGT TCT ATG A-3′; Sm RV 5′-CAC GC TCT CG C AAA TAA TCT AAA-3′; Sm probe 5′-[FAM] TCG TTG TAT CTC CGA AAC CAC TGG ACG [(BHQ1])-3′ all synthesized by Eurofins Genomics, Ebersberg, Germany.

The 25 µl reaction mix contained 2.5 µl DNA, 1 × QuantiFast Pathogen Master Mix (QuantiFast ® Pathogen PCR + IC Kit, Qiagen, Hilden, Germany), 400 nmol/L of each Sm Primer and 200 nmol/L of Sm probe. The PCR runs consisted of an initial step of 5 min at 95 °C followed by 40 successive cycles of 15 s at 95 °C and 30 s at 60 °C. The reaction was run on the StepOne real-time PCR system (Applied Biosystems). DNA detection was expressed by cycle threshold (Ct)-values. In every run, the non-template control was negative (Ct = 0), the positive control (*S. mansoni* egg DNA) was positive (Ct < 22) and the internal control to test successful amplification and to exclude the presence of PCR inhibitors was positive (Ct < 33). A test was considered positive when the threshold was attained within 40 PCR cycles (Ct < 40). Each sample was tested only once, not in replicates.

### Statistical analyses

Statistical analysis were carried out using IBM SPSS Statistics version 24 (SPSS Inc., Chicago, USA) and Microsoft Excel 2013 (Microsoft Corporation, Redmond, USA). A *P*-value lower than 0.05 was considered statistically significant.

The prevalence of *S. mansoni* was determined for each diagnostic method. The arithmetic mean egg count was calculated as the average egg count of the two KK smears, and classes of intensity of *S. mansoni* were determined as light (1–99 EPG), moderate (100–399 EPG) and heavy (> 400 EPG). The thresholds are set according to the values published by the WHO [41]. Medians were calculated for the Ct-values. This study used a combined diagnostic reference of positive results by serum-based real-time PCR and positive egg counts as determined by KK. A combined reference is used to obtain a reliable result and has been described in other studies [42, 43]. Since this approach assumes a specificity of 100% for both test methods, only sensitivity and negative predicted value (NPV) were calculated for the assays used. Diagnostic results were converted to binary variables (1 = positive and 0 = negative). Kappa coefficient (κ) was used to statistically estimate the agreement between one diagnostic tool compared to the reference. Differences in the positivity rate between our reference and the other used methods to diagnose schistosomiasis were determined using the McNemar test.

By using the real-time PCR method, the Ct-value is detected. The Ct is defined as the number of cycles required for the fluorescent signal to cross the threshold (i.e. exceeds background level). Ct levels are inversely proportional to the amount of target nucleic acid in the sample (the lower the Ct level the greater the amount of target nucleic acid in the sample). A nonparametric test (Wilcoxon signed-rank test) was used to compare the median Ct-values of the two different methods.

## Results

### Demographic information of participants

Study participants who provided sample material for the current study consisted of 76 females and 24 males with a mean age of 35.6 years (range: 17 to 70 years). Of the 100 study participants, two did not provide stool samples and five participants did not provide blood for serum collection. Specimens from 93 individuals were investigated by all methods.

### Prevalence of *Schistosoma mansoni* using KK technique and POC-CCA test

Microscopic stool examination by KK method showed eggs of *S. mansoni* in 43.9% of the samples. Egg loads varied between 12 and 1248 EPG with a median of 36 EPG. The prevalence determined with the commercially available POC-CCA rapid test was 80%.

### Prevalence of *Schistosoma mansoni* based on DNA detection using PCR

**Fig. 1:**
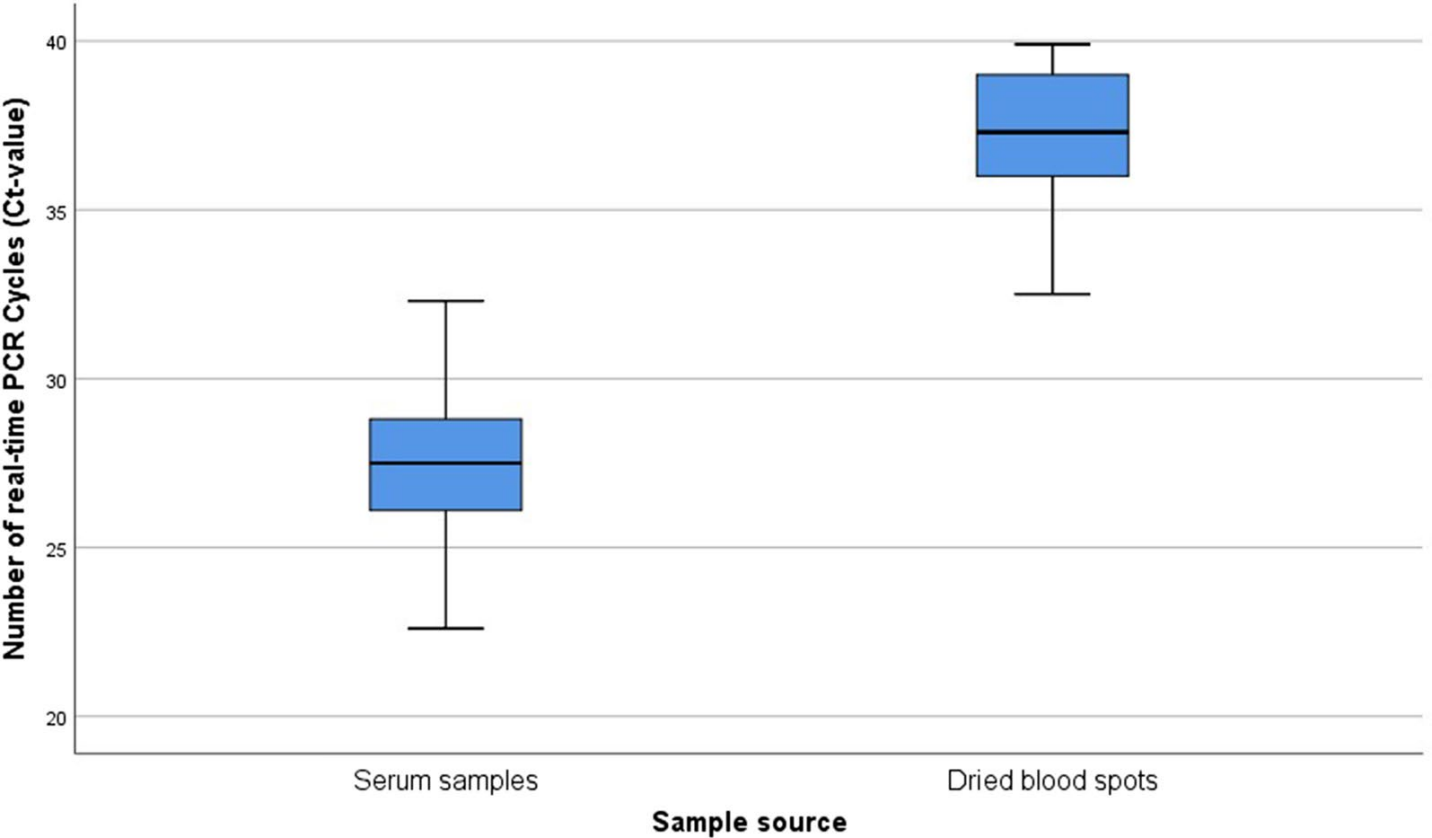
Boxplot of 37 paired real-time PCR positive samples comparing the distribution of Ct-values in serum samples (median Ct-value: 27.5) and dried blood spot cards (median Ct-value: 37.3)

Real-time PCR detected *S. mansoni* DNA in 88.4% of the serum samples with Ct-values ranging between 22.6 and 37.4 and a median Ct-value of 30.1. In DBS, real-time PCR yielded 41% *S. mansoni* positive samples with Ct values between 32.5 and 39.9 and a median Ct-value of 37.2 (Table 1). Comparing the 37 samples, which were positive by serum-based and DBS-based real-time PCR, showed that the Ct values were significantly higher in the DBS (median: 37.3, minimum: 32.5, maximum: 39.9) than in the serum samples (median: 27.5, minimum: 22.6, maximum: 33.6) of the same study participants (*P* < 0.001) (Fig. 1).

**Table 1:** Prevalence of *Schistosoma mansoni* and median (minimum, maximum) of positive results for the different diagnostic tests

Method	Cases/total	%	EPG (Kato-Katz)/Ct-values real-time PCR		
			Median	Minimum	Maximum
Microscopy (Kato-Katz)	43/98	43.9	36	12	1248
POC-CCA	80/100	80.0			
Serum real-time PCR	84/95	88.4	30.1	22.6	37.4
DBS real-time PCR	41/100	41.0	37.2	32.5	39.9

*Ct* cycle threshold, *DBS* dried blood spot, *EPG* eggs per gram, *PCR* polymerase chain reaction, *POC-CCA* point-of-care circulating cathodic antigen

A combination of the parasitological KK test and real-time PCR of serum samples was used as reference. According to this procedure, 92.5% (86/93) *S. mansoni* positive samples were determined. Serum-based real-time PCR displayed the highest sensitivity (95.4%) and NPV (63.6%), as well as high Kappa agreement (k = 0.755, *P* < 0.001). This technique missed 4 out of 86 positive cases. The lowest sensitivity (45.4%) and NPV (13%) was achieved by the method of DBS-based real-time PCR. The positivity rate obtained from the combination of KK and serum-based real-time PCR was statistically higher than that obtained from microscopy (*P* < 0.001), the POC-CCA test (*P* = 0.002) or DBS-based real-time PCR (*P* < 0.001). In addition, there was a poor agreement among KK or DBS real-time PCR and the reference. These results are summarized in Table 2.

**Table 2:** Sensitivity, negative predicted value (NPV) with confidence intervals (*CI*), McNemar and Kappa statistic of the different diagnostic tests used

Test method	Sensitivity (95% *CI*)	NPV (95% *CI*)	*P* McNemar	Kappa (*P* value)
Microscopy (KK)	48.8% (37.9–59.9)	13.7% (5.7–26.3)	< 0.001	0.126 (*P* = 0.013)
POC-CCA	84.9% (75.5–91.7)	31.6% (12.6–56.6)	= 0.002	0.395 (*P* < 0.001)
Serum-based real-time PCR	95.4% (88.5–98.7)	63.6% (30.8–89.1)	= 0.125	0.755 (*P* < 0.001)
DBS real-time PCR	45.4% (34.6–56.5)	13% (5.4–24.9)	< 0.001	0.111 (*P* = 0.019)

Serum-based real-time PCR and Kato-Katz results were used as reference (any positives by either of these two methods were deemed to be true positive)

*DBS* dried blood spot, *KK* Kato-Katz, *PCR* polymerase chain reaction, *POC-CCA* point-of-care circulating cathodic antigen

Of the 42 KK-positive participants, of whom complete sample sets were available, 29 (69%) showed light, 6 (14.3%) moderate and 7 (16.7%) heavy *S. mansoni* infection intensities. When comparing the sensitivities of the individual test methods with regard to the different infection intensities, it was shown that the sensitivity of all methods increased with increasing egg counts. Both the POC-CCA test and the serum-based real-time PCR showed a sensitivity of 100% for medium and severe infections. The DBS real-time PCR showed a sensitivity of only 85.7% even for severe infections (Table 3). Of the 56 KK-negative samples, 37 were positive (66.1%) using the POC-CCA test and 12 were positive (21.4%) using the DBS-PCR. Serum PCR detected 45 of 52 (86.5%) KK-negative samples as positive.

**Table 3:** Sensitivity with confidence intervals (*CI*) of the different diagnostic tests compared to *Schistosoma mansoni* infection intensities [classified according to WHO guidelines using the Kato-Katz (KK) method [41]]

Test method	Sensitivity (95% *CI*)		
	KK 1–99 EPG (*n* = 29)	KK 100–399 EPG (*n* = 6)	KK > 399 EPG (*n* = 7)
POC-CCA	96.6% (82.2–99.9)	100% (54.1–100)	100% (59.0–100)
Serum-based real-time PCR	89.3% (67.3–96)	100% (54.1–100)	100% (59.0–100)
DBS real-time PCR	65.5% (45.7–82.1)	66.7% (22.3–95.7)	85.7% (42.1–99.6)

*DBS* dried blood spot, *EPG* eggs per gram, *KK* Kato-Katz, *POC-CCA* point-of-care circulating cathodic antigen

## Discussion

Numerous studies in recent years have shown that PCR-based techniques are highly specific and sensitive for the diagnosis of schistosomiasis. However, these assays are rarely used in *Schistosoma* endemic countries, as it requires expensive equipment and highly skilled laboratory personnel [44]. In addition, in remote tropical areas, sampling, transport and storage of venous blood samples are often sub-optimal [29]. In our study, we investigated the value of detecting cell-free *S. mansoni* DNA in serum and DBS as an alternative sample source compared to the classical diagnostic methods for the detection of eggs in stool or the detection of schistosome antigens in urine. Most infections with *S. mansoni* were detected by serum-based real-time PCR (88.4%), followed by the POC-CCA test (80.0%). Comparatively few infections were identified using the DBS-based real-time PCR method (41.0%) and the microscopic KK method (43.9%). In the study by Espírito-Santo et al. in a low endemic setting, it was also shown that the positivity rate of serum-based real-time PCR was higher than that obtained using parasitological tests [45]. To determine diagnostic accuracy, the combined results of microscopy and serum-based real-time PCR were used as a reference in this study, with both methods considered 100% specific. This procedure has also been reviewed and applied in other studies [42, 46]. The serum-based real-time PCR showed excellent performance with a sensitivity of 95.4%, while DBS-based real-time PCR showed the lowest sensitivity (45.4%). The sensitivity of the serum-based real-time PCR was also higher than that of the POC-CCA test (95.4% vs 84.9%) when considering the total collective.

According to WHO guidelines, *S. mansoni* infection intensities can be classified on the basis of KK results [41]. The majority of the KK-positive study participants (69.0%) showed light infection intensities. In this study the highest sensitivity was found for the POC-CCA method (96.6%) followed by serum-based real-time PCR (89.3%). Thus, despite the microscopic findings of eggs, no *S. mansoni* infection could be detected in some serum samples with real-time PCR. Possibly the samples were mixed up or incorrectly labelled, and urine and blood samples were not taken from the same participant. However, false negative results of PCR methods have been reported by different research groups [47, 48]. One possible cause could be degradation of DNA during transport from the field or during storage of the samples [49].

Observation of the Ct-values showed that these were significantly higher in DBS (median: 37.3) than in serum samples (median: 27.5, *P* < 0.001), which reflects a lower parasite-specific DNA load on the filter cards. This discrepancy could be due to the small amount of blood applied to the DBSs and the small volume used for the extraction process, which might lead to a higher rate of false negative results. A 6 mm diameter punch contains approximately 8.7 ± 1.9 μl of spotted blood, so it can be assumed that the concentration of genetic material is low [29]. To increase the volume of the analyzed blood eight punches were used in this study. Nevertheless, DBS-based real-time PCR did not perform well in our study. Similar results have also been observed in various malaria studies. In Tanzania, Strøm et al. found almost two times more positive results when a protocol with 200 μl venous blood was used: The positivity of the PCR was 24.5% when using 200 μl whole blood and 11.2% when using DBS [50]. The study by Canier et al. comparing malaria PCR detection from DBS and venous blood samples showed no significant difference in the detection rates of malaria parasites. False-negative results were obtained when using DBS, but these were *Plasmodium vivax* infections, which are often found in asymptomatic individuals with very low parasite density [51]. Possibly the sensitivity of this method could be increased by using other extraction methods or two sequential amplifications by PCR. Promising results have already been obtained by Espírito-Santo et al. on the detection of *S. mansoni* in stool samples by PCR [52]. In addition, the data from several studies suggest that there may be differences between filter papers from different manufacturers and that further studies should be carried out to evaluate several types of filter paper [53–55].

DBS cards have been used in numerous studies over the last five decades and have proven to be a simple and well-accepted sampling tool [55]. The collection of DBS is simple, as only a finger prick is required and no venipuncture [28, 29]. Furthermore, DBS require simpler storage and transport conditions, do not need a cold chain, and allow retrospective PCR analysis [28, 56]. Nevertheless, DBS did not demonstrate to be a valuable alternative sample source in the present study and could not simplify the applicability of DNA-based diagnosis of schistosomiasis in the field.

This study was not carried out without limitations. Given the lack of a diagnostic standard, two test methods were combined in the current work to form an artificial reference. As this implied that the tests were evaluated to a certain extent on the basis of their own results, only a limited interpretation of the data is possible. Also, it is known that degradation of DNA in DBS can occur during storage and extraction and affects the sensitivity of diagnostic assays [56–59]. Therefore, further studies with different storage conditions (humidity and temperature), storage duration and different types of filter paper should be performed to determine in which way this affects the results of DBS-based real-time PCR.

## Conclusions

The results of the DBS-based real-time PCR in our study do not suggest the use of DBS cards as an alternative sample source for the diagnosis of *S. mansoni*. Extensive testing must be done before filter cards are used in future studies to detect circulating DNA from *Schistosoma* by real-time PCR. In addition, our results showed that the POC-CCA test is a valuable, easy to use and accurate test for detecting *S. mansoni* infections in moderate or highly endemic areas. There were only minor differences compared to the serum-based real-time PCR. Even in areas with low prevalence and intensity, the prevalence determined by POC-CCA is higher than that of KK and provides a sensitive and accurate screening tool for *S. mansoni*.

## Data Availability

All relevant data are within the paper and its Supporting Information files.

## Abbreviations

CCA: Circulating cathodic antigen
*CI*: Confidence intervals
Ct: Cycle threshold
DBS: Dried blood spot
EDTA: Ethylenediaminetetraacetic acid
EPG: Eggs per gram
KK: Kato-Katz
MDA: Mass drug administration
NIMR: National Institute for Medical Research
NPV: Negative predicted value
PBS: Phosphate-buffered saline
PCR: Polymerase chain reaction
POC-CCA: Point-of-care circulating cathodic antigen
PZQ: Praziquantel
WASH: Water, sanitation and hygiene
WHO: World Health Organization
